# The soluble VEGF receptor sFlt-1 contributes to endothelial dysfunction in IgA nephropathy

**DOI:** 10.1371/journal.pone.0234492

**Published:** 2020-08-13

**Authors:** Yaling Zhai, Youxia Liu, Yuanyuan Qi, Xiaoqing Long, Jingge Gao, Xingchen Yao, Yazhuo Chen, Xinnian Wang, Shan Lu, Zhanzheng Zhao

**Affiliations:** 1 The Renal Research Institution of Zhengzhou University, Department of Nephrology, the First Affiliated Hospital of Zhengzhou University, Zhengzhou, China; 2 Department of Nephrology, Tianjin Medical University General Hospital, Tianjin, China; Center for Molecular Biotechnology, ITALY

## Abstract

Endothelial injury is a common manifestation in IgA nephropathy (IgAN). After the previous identification of the upregulated soluble fms-like tyrosine kinase-1 (sFlt-1) correlated with endothelial injury in IgAN, in the present study, we further explored the role of sFlt-1 in endothelial injury in IgAN. We enrolled 72 patients with IgAN and detected the sFlt-1 levels. The polymeric IgA1 (pIgA1) complexes were isolated from the pooled plasma samples of another 10 patients with IgAN. Apoptosis proteins were detected in cultured human umbilical vein endothelial cells (HUVECs) with the stimulation of recombinant sFlt-1 or the caspase-9 inhibitor Z-LEHD-FMK. We identified there were positive correlations between sFlt-1 and IgA-IgG complex as well as vWF levels in patients with IgAN. The sFlt-1 levels in HUVECs were significantly upregulated by pIgA1 complex derived from IgAN patients in a concentration-dependent manner. The proliferation ability of HUVECs was damaged when stimulated with sFlt-1 protein in a time- and dose- dependent manner. And the apoptosis rate was up-regulated significantly as the stimulation concentrations of sFlt-1 increased. We found sFlt-1 challenge could significantly increase the expression of vWF. In addition, sFlt-1 increased the levels of caspase-9, caspase-3, Bax and mitochondrial membrane potential; facilitated the release of cytochrome C from mitochondria to cytoplasma. In contrast, Z-LEHD-FMK attenuated high sFlt-1-induced HUVECs apoptosis. In conclusion, our study demonstrated that sFlt-1 expression was up-regulated by the challenge of pIgA1 complex derived from patients with IgAN. Furthermore, increased sFlt-1 facilitated human umbilical vein endothelial cells apoptosis via the mitochondrial-dependent pathway.

## Introduction

Immunoglobulin A nephropathy (IgAN) is the most common type of primary glomerulonephritis worldwide, with approximately 10–20% of patients progress to end-stage renal disease within 20 years [1, 2]. The pathogenesis of IgAN remains unclear. More and more evidence indicated that circulating polymeric IgA1 (pIgA1) immune complexes played an important role in the initiation of kidney injury in IgAN [3, 4]. Endothelial cells are the first layer of cells exposed to damage induced by hemodynamic or immunologic insults. Recently, Kusano et al reported the loss of endothelial cells occurred in IgAN and may contribute to the progression of IgAN [5]. They also pointed out up to 50% thrombotic microangiopathy (TMA) lesions occurred in normotensive patients with near-normal renal histology. Many studies showed that plasma von Willebrand Factor (vWF) and vasoconstrictor endothelin-1 (ET-1), specific markers for endothelial cells injury, were elevated in patients with IgAN [6, 7]. Therefore, vascular endothelial injury was regarded as a major contributor to glomerular injury in IgAN.

Soluble fms-like tyrosine kinase-1 (sFlt-1), a vascular endothelial growth factor (VEGF) antagonist, has been suggested as a marker of endothelial dysfunction in preeclampsia [8, 9]. Many studies demonstrated excess sFlt-1 was associated with endothelial dysfunction in patients with chronic kidney disease (CKD) [10, 11]. Our previous study reported sFlt-1 level elevated in IgAN patients and also correlated with proteinuria, hypertension and vWF level [12]. These results suggested that elevated sFlt-1 contributed to endothelial injury in IgAN. However, the mechanism that leads to this dysfunction remains unclear.

The mitochondrial pathway is considered a mechanism to induce apoptosis in human umbilical vein endothelial cells (HUVECs) and glomerular endothelial cells [13, 14]. The mitochondrial cell death pathway commences when apoptogenic molecules induced an increased ratio of pro-apoptotic Bax/anti-apoptotic B-cell lymphoma 2 (Bcl-2), followed by the change of mitochondrial outer membrane permeabilization. This process resulted in a significant increase in the release cytochrome C from mitochondria, an activation of caspases and subsequently apoptosis. Whether sFlt-1 induces endothelial injury by triggering the mitochondrial pathway remains to be investigated.

In this study, we sought to understand the mechanism of endothelial injury induced by sFlt-1 in IgAN. We detected sFlt-1 levels using pIgA1 complex derived from patients with IgAN. Furthermore, we analyzed the expression of mitochondrial-dependent apoptosis-related proteins in HUVECs stimulated with recombinant sFlt-1 protein and specific protein-kinase inhibitor. The findings identified that sFlt-1 could induce apoptosis in HUVECs through the mitochondrial-dependent pathway in IgAN for the first time.

## Materials and methods

### Study population

Serum samples were collected after obtaining written informed consent from 72 patients with primary IgAN diagnosed between 1^st^ January to 1^st^ July of 2018 in the First Affiliated Hospital of Zhengzhou University. The diagnosis of IgAN was based on the presence of IgA deposition in the glomerular mesangium by immunofluorescence and electron-dense material deposition in the mesangium by electronic microscopy. The exclusion criteria included patients with Henoch-Schönlein purpura and other secondary IgAN diagnosed by detailed clinical and laboratory examinations. The study protocol was reviewed and approved by the Ethics Committee of the First Affiliated Hospital of Zhengzhou University. Informed consent was obtained from all included patients.

### Plasma IgA and IgA-IgG complex

For recruited IgAN patients, 10 mL EDTA anticoagulated peripheral venous blood was extracted on the day of the renal biopsy. Total IgA (EMD Chemicals, USA) levels were determined by ELISA, as previously reported [15]. For IgA-IgG complexes, 10 μg/ml F(ab’)_2_ fragment of goat IgG specific for human IgA (Jackson Immuno-Research Labs, USA) was coated with microtitration plates as described. Plasma was then added and incubated with horseradish peroxidase-labeled rabbit anti-human IgG (Sigma, USA). The optical density was measured at 450/570 nm. The immune complex levels were expressed as the ratio of the optical densities of the sample to that of the positive control [15].

### Isolation of pIgA1 complex

The pIgA1 complexes were isolated from the pooled plasma samples of another ten patients with IgAN using jacalin affinity chromatography, as previously reported [16]. Briefly, the pooled plasma was diluted 1:2 with phosphate buffered saline (PBS), and then applied to a jacalin affinity chromatography column (Vector Laboratories, USA). The column was washed with Tris-HCl (pH 7.4), and IgA1 was eluted with 0.1 M melibiose (Sigma, USA) in PBS. The eluted fractions were pooled and concentrated by ultrafiltration using Vivaspin (Sartorius Stedim Biotech, Germany). The purified pIgA1 was separated by molecular sieve chromatography using a Sephacryl S-300 gel filtration column (GE Healthcare Life Sciences, Sweden). The pIgA1 from IgAN patients was identified by Western blotting.

### Cell culture experiments

HUVECs were purchased from Sciencell Research Laboratories (USA). The cells were cultured according to the manufacturer’s instructions in Endothelial Cell Medium (ECM; Sciencell, USA) with 10% fetal bovine serum (FBS), endothelial cell growth supplement, 100 μg/mL streptomycin and 100 U/mL penicillin at 37°C in a humidified atmosphere with 5% CO_2_ and 95% O_2_. The culture medium was replaced every 2 days or as necessary. HUVECs were seeded into 6-well plates at a density of 5 × 10^5^ cells per well. After overnight serum starvation, HUVECs were incubated with pIgA1 complex at final concentrations of 0, 100, 400 ug/ml from IgAN patients for 24h to determine the expression of sFlt-1 in cell supernatant. To evaluate the effects of sFlt-1 in the process of HUVECs injury, HUVECs were stimulated with/without the caspase-9 inhibitor Z-LEHD-FMK (2μmol/L, Apexbio, USA) for 1 h, then stimulated with recombinant sFlt-1 in different concentrations (0 ng/mL, 100 ng/mL, 1000 ng/mL) for 24h. After centrifugation, the supernatants of the cultured HUVECs and the cells were collected.

### CCK-8 assay

Cell viability was assessed using a cell counting kit-8 (CCK-8). Cells were seeded in 96-well plates and cultured in ECM at different concentration of sFlt-1 for 24h and 48h. 10ul CCK8 solution was added into each well, followed by incubated for 2 h at 37°C. The cell viability was revealed by the relative absorbance which was measured at 450 nm.

### Annexin V-FITC/PI staining

Apoptotic cells were detected by staining with Annexin-V-FITC and propidium iodide (PI) Apoptosis Detection Kit (BD, USA). The cell cycle stage measured by flow cytometry instrument (Becton-Dickinson, USA). The Annexin V-FITC−/PI− cells were considered to normal healthy cells. Cells stained with Annexin V-FITC+/PI− were regarded as early apoptosis cells. The Annexin V-FITC+/PI+ cells were regarded as a measure of late apoptosis. The Annexin V-FITC−/PI+ cells were considered as necrotic cells. Both early (Annexin V-FITC+/PI−) and late (Annexin V-FITC+/PI+) apoptotic cells were included in the cell death analyses.

### TUNEL staining

Terminal deoxynucleotidyl transferase-dUTP nick-end labeling (TUNEL) staining kit was used to evaluate apoptotic cells (Roche, Switzerland) according to the manufacturer's introductions. At the end of treatment of sFlt-1 in different concentrations, HUVECs were washed twice with PBS, fixed with 4% paraformaldehyde for 30 min, and permeabilized with Triton X-100 for 15 min. TUNEL-positive cells were observed with a fluorescence microscope. The apoptotic index was expressed as the percentage of TUNEL positive cells. Three independent experiments were then averaged and statistically analyzed.

### Western blot analysis

The HUVECs were collected, lysed and concentrated for protein extraction. Thirty ug of protein samples were separated using 10% sodium dodecyl sulfate-polyacrylamide gel electrophoresis for 2h and then transferred to polyvinylidene difluoride membranes for 1.5h. The membranes were blocked with 5% nonfat milk, and incubated overnight at 4°C with the primary antibody below. Western blot analysis was performed using the following antibodies: Bcl-2, Bax, cytochrome C, caspase-3, cleaved Caspase-9, COX IV and β-actin (Cell Signaling Technology, USA). Subsequently, secondary antibodies (Zhongshan Inc, China) were added and incubated for 2 h. The signals were visualized after adding enhanced chemiluminescence (Pierce, USA). Membranes were visualized using the Amersham Imager 600 system.

### Detection of sFlt-1 and vWF

The plasma and cell culture supernatants of vWF (DAKO, Denmark) and plasma sFlt-1 (R&D Systems, USA) were detected using commercial ELISA kits according to the manufacturer’s specifications.

### Mitochondrial membrane potential assay

The mitochondrial membrane potential (Δψm) was detected by the JC-1 assay kit (Molecular Probes, USA). Following treatment with sFlt-1, HUVECs were incubated with JC-1 for 30 min at 37°C and transferred to a 96-well plate. The red and green fluorescence were captured using a fluorescence plate reader. The changes in the ratio of red (excitation/emission, 550/600 nm) to green fluorescence (excitation/emission, 485/535 nm) was calculated in triplicates.

### Statistical analysis

All data were analyzed using SPSS 19.0 software (SPSS, USA). The data were expressed as means and SD from at least 3 independent experiments. One-way analysis of variance (ANOVA) test was used in the experiments that had three or more groups to determine the significant differences between the treatment and control groups. Statistical comparisons of the results were made using 2-tailed Student’s t-test. Correlation analysis was performed by Pearson′s correlation test. *p* < 0.05 was considered statistically significant.

## Results

### The correlation between sFlt-1 and IgA-IgG complex, as well as vascular injury biomarker

We first examined the correlations between sFlt-1 and IgA-IgG complex, as well as vascular injury biomarker of vWF by Pearson′s correlation test. Positive correlations were observed between sFlt-1 and IgA-IgG complex (r = 0.466, *p* < 0.001; Table 1) as well as vWF levels (r = 0.397, *p* = 0.001; Table 1) in patients with IgAN. However, there was no significant correlation between IgA-IgG complex and vWF levels (r = 0.034, *p* = 0.775; Table 2).

**Table 1:** The correlations between sFlt-1 and IgA-IgG complex, as well as vWF.

	sFlt-1(pg/ml)	IgA-IgG complex/IgA (%)	vWF (ng/ml)
sFlt-1 (pg/ml)		r = 0.466, p < 0.001	r = 0.397, p = 0.001
IgA-IgG complex/IgA (%)	r = 0.466, p < 0.001		r = 0.034, p = 0.775

**Table 2:** The baseline data for patients with IgAN.

Characters	Value
Gender (male, %)	40 (55.56%)
Age (year, mean ± SD)	35.39 ± 11.34
Serum creatinine (μmol/L, median, IQR)	78.50 (63,135)
Uric acid (μmol/L, mean ± SD)	349.23 ± 106.28
Serum albumin (g/L, median, IQR)	37 (32.60, 40.90)
24h urine protein (g/d, median, IQR)	1.97 (1.09, 3.70)
Hypertension (n, %)	25 (34.72%)
Oxford classification	
M1 (n, %)	14 (19.44%)
E1 (n, %)	20 (27.78%)
S1 (n, %)	53 (73.61%)
C1/C2 (n, %)	10 (13.89%)/ 2 (2.78%)
T1/T2 (n, %)	10 (13.89%)/ 16 (22.22%)
Arteriole injury (n, %)	46 (63.89%)
Prednisone or immunosuppressor (n, %)	37 (51.39%)

### pIgA1 immune complex derived from IgAN patients upregulated sFlt-1 expression in HUVECs

**Fig 1:**
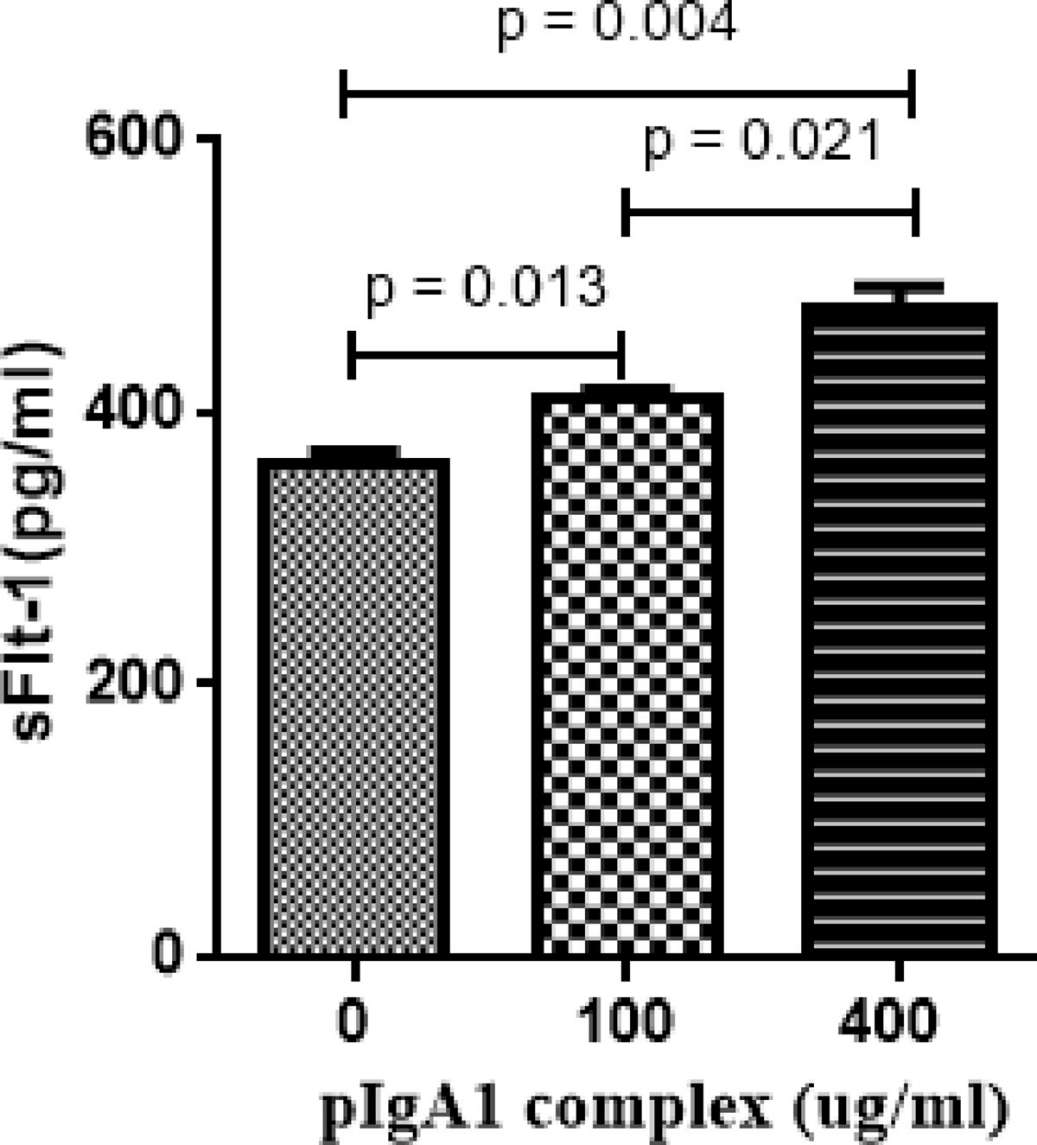
The sFlt-1 expression was upregulated by pIgA1 complex in the supernatant of HUVECs. The HUVECs were treated with pIgA1 complex. The histogram showed that sFlt-1 expression was increased significantly by pIgA1 complex derived from IgAN patients in a concentration-dependent manner. Results are the means ± standard deviation of three independent experiments. Statistically significant at *p* < 0.05 level vs. without pIgA1-treatment HUVECs.

We detected the sFlt-1 levels in culture supernatant of HUVECs under pIgA1 complex challenge. Our results indicated that the sFlt-1 levels were significantly upregulated by pIgA1 complex derived from IgAN patients in a concentration-dependent manner (100 ug/ml vs. 0 ug/ml: 409.04 ± 11.96 vs. 362.59 ± 14.53 pg/ml, *p* = 0.013; 400 ug/ml vs. 0 ug/ml: 475.44 ± 28.67 vs. 362.59 ± 14.53 pg/ml, *p* = 0.004; 400 ug/ml vs. 100 ug/ml: 475.44 ± 28.67 vs. 409.04 ± 11.96 pg/ml, *p* = 0.021, Fig 1).

### The proliferation ability of HUVECs was damaged when stimulated by sFlt-1 protein in a time- and dose- dependent manner

**Fig 2:**
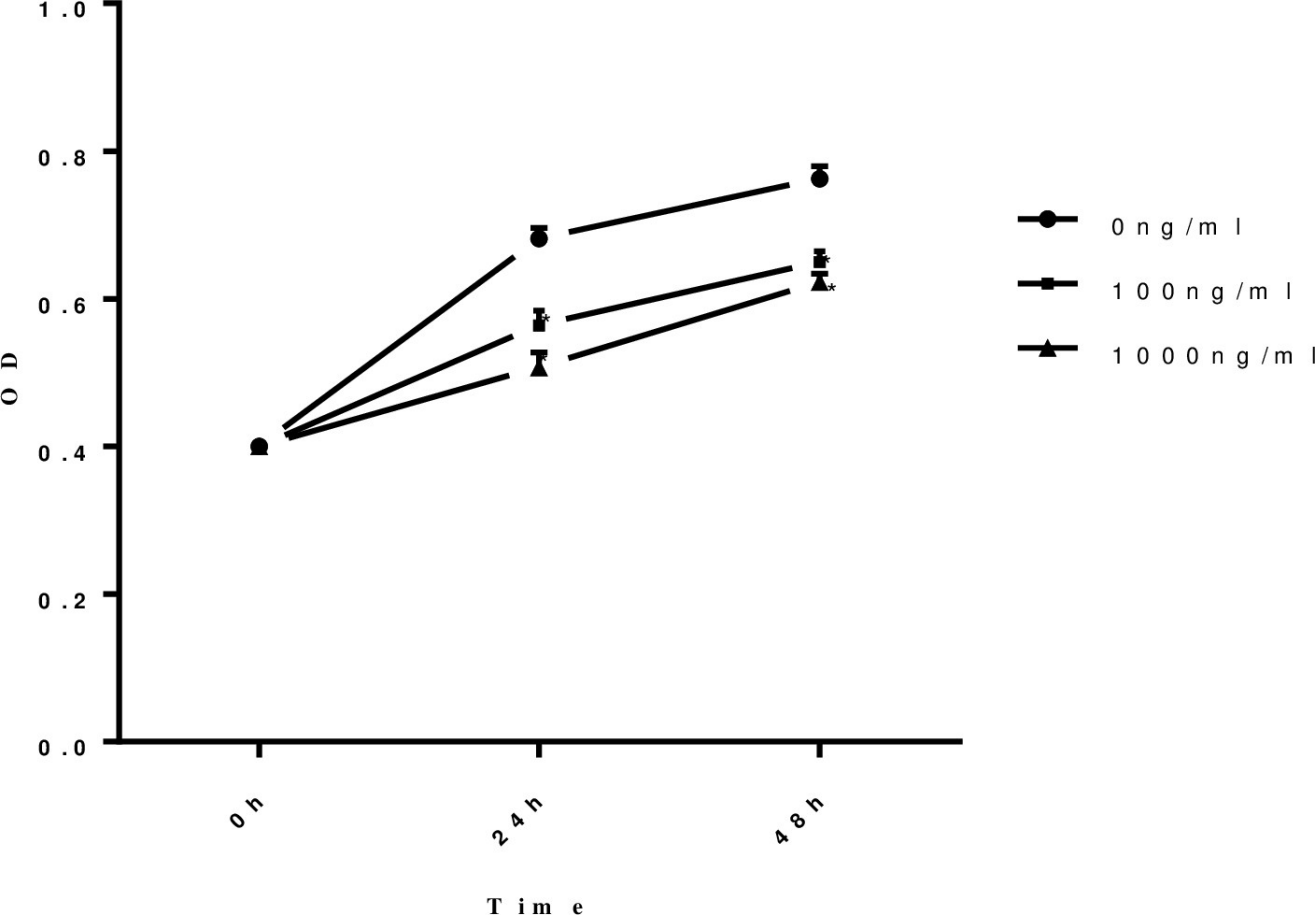
The proliferation ability of HUVECs was damaged when stimulated by sFlt-1 protein in a time and dose dependent manner. The proliferation ability of HUVECs was measured by CCK-8 after stimulated by different concentration of sFlt-1 at different time points. The proliferation of HUVECs was damaged when stimulated by sFlt-1 protein in a time- and dose- dependent manner. Circles with solid lines represent the proliferation ability in HUVECs untreated with sFlt-1, squares with solid lines represent the proliferation ability in HUVECs treated with 100ng/ml sFlt-1, and triangles with solid lines represent the proliferation ability in HUVECs treated with 1000ng/ml sFlt-1. Results are the means ± standard deviation of three independent experiments. Asterisks indicate a significant difference from the untreated group (*p* < 0.05).

The proliferation ability of HUVECs was measured by CCK-8 after stimulated by different concentrations of sFlt-1 protein (0 ng/ml, 100 ng/ml and 1000 ng/ml) at different time points (0h, 24 h and 48 h) to evaluate the involvement of sFlt-1 in the process of HUVECs injury in IgAN. The results showed the proliferation ability of HUVECs was damaged when stimulated by sFlt-1 protein in a time- and dose- dependent manner (Fig 2).

### The levels of vascular injury marker were increased as the sFlt-1 stimulated

**Fig 3:**
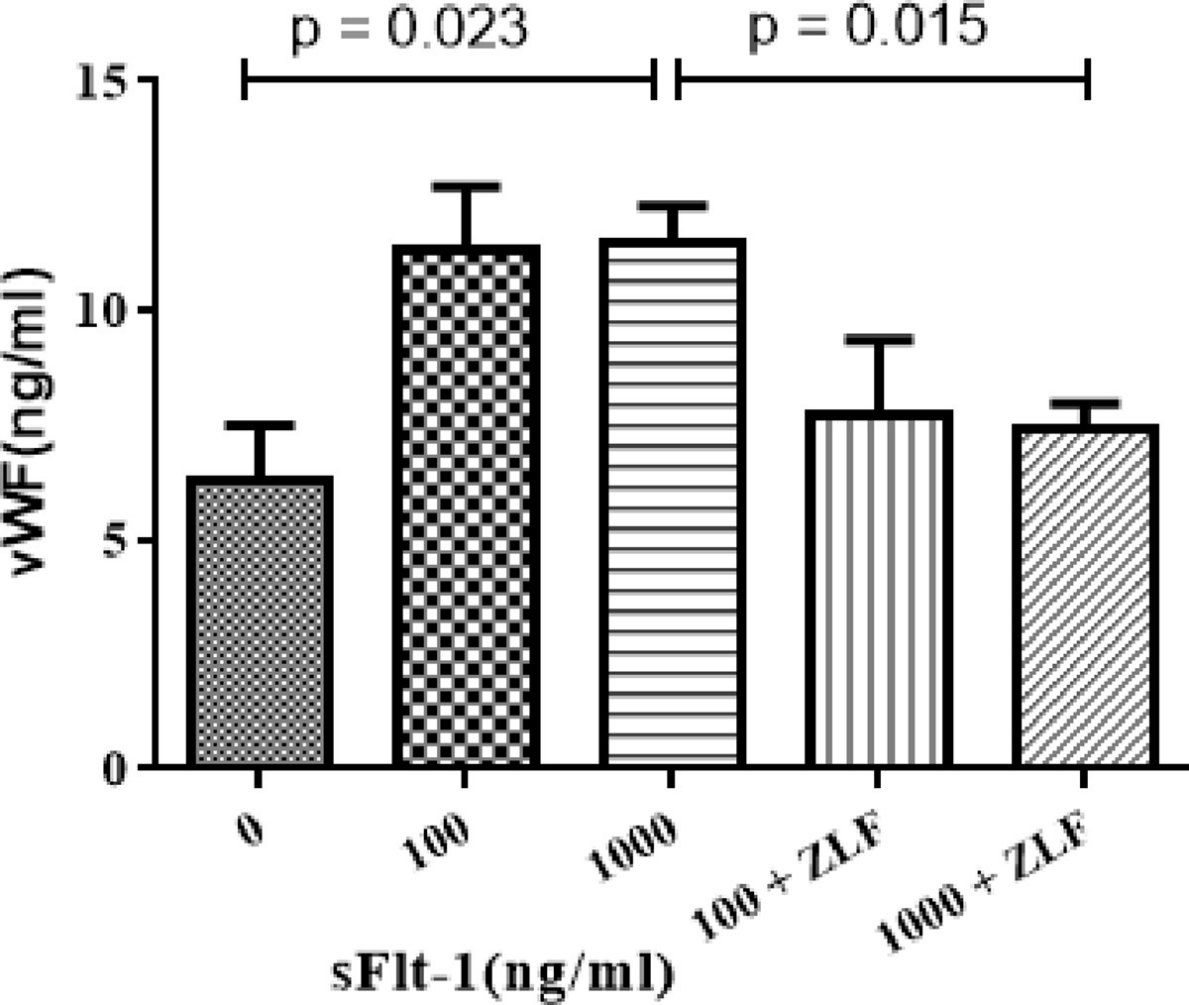
Effects of sFlt-1 and Z-LEHD-FMK on the vascular injury marker of vWF. The HUVECs were treated with the indicated concentrations of sFlt-1, and then treated with or without Z-LEHD-FMK. The histogram showed that vWF expression were significantly upregulated by sFlt-1 stimulation at 1000 ng/ml (11.50 ± 1.39 vs. 6.31 ± 2.07 ng/ml, *p* = 0.023) compared with those of untreated cells, whereas the expression of vWF was down-regulated after added with Z-LEHD-FMK (11.50 ± 1.39 vs. 7.39 ± 1.78 ng/ml, *p* = 0.015). Results are the means ± standard deviation of three independent experiments.

We detected the levels of vWF, a well-known marker of endothelial dysfunction, in culture supernatant under sFlt-1 challenge. Our results indicated that the expression of vWF was significantly upregulated by sFlt-1 stimulation at 1000 ng/ml (11.50 ± 1.39 vs. 6.31 ± 2.07 ng/ml, *p* = 0.023; Fig 3) compared with that of untreated cells.

### The apoptosis rate was up-regulated significantly as the stimulation concentration of sFlt-1 increased

**Fig 4:**
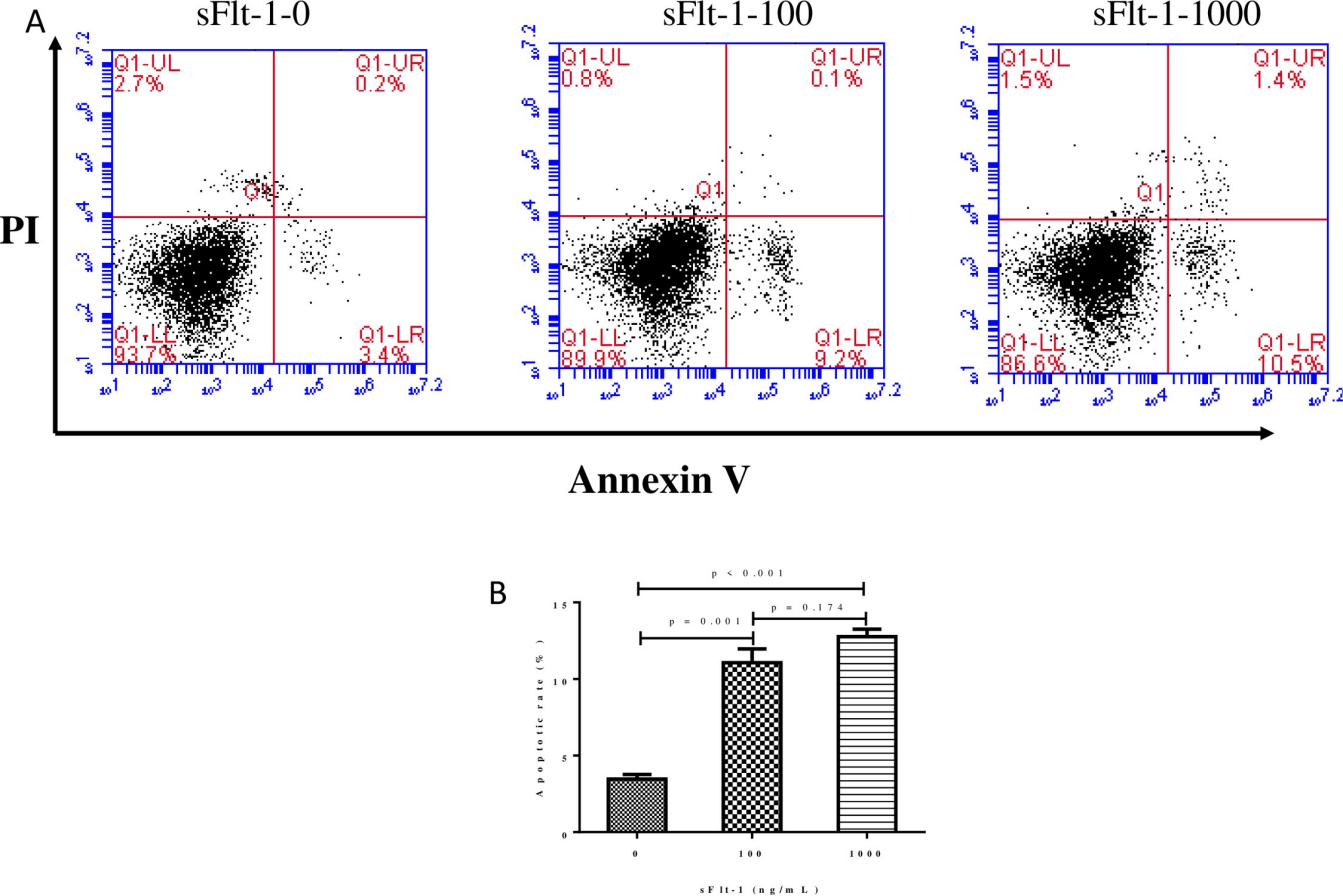
The apoptosis rate of HUVECs was measured using an Annexin V-APC apoptosis analysis kit. The apoptosis of HUVECs was detected by flow cytometry. The figure showed a representative set of the dot-plot graph of flow cytometry analysis of the following groups: A: the indicated concentrations of sFlt-1-treated group, and B: statistical result of apoptosis rate of HUVECs in different groups. The apoptosis rate was up-regulated significantly as the stimulation concentrations of sFlt-1 increased (100 ng/ml vs. 0 ng/ml, 11.07 ± 1.57% vs. 3.47 ± 0.51%, *p* = 0.001; 1000 ng/ml vs. 0 ng/ml 12.77 ± 0.85% vs. 3.47 ± 0.51%, *p* < 0.001). There was no obvious difference between cells with 1000 ng/ml and 100 ng/ml (12.77 ± 0.85% vs. 11.07 ± 1.57%, *p* = 0.174). Results are the means ± standard deviation of three independent experiments.

Two quantitative evaluations, flow cytometry using Annexin V-FITC/PI staining and TUNEL, were adopted to assess the apoptosis rate. The flow cytometry revealed the apoptotic rate increased markedly when HUVECs exposed to sFlt-1 compared with those without treatments. And the apoptosis rate was up-regulated significantly as the stimulation concentration of sFlt-1 increased (100 ng/ml vs. 0 ng/ml, 11.07 ± 1.57% vs. 3.47 ± 0.51%, *p* = 0.001; 1000 ng/ml vs. 0 ng/ml 12.77 ± 0.85% vs. 3.47 ± 0.51%, *p* < 0.001). There was no obvious difference in apoptosis rate between the intervention with sFlt-1 at 1000 ng/ml and 100 ng/ml (12.77 ± 0.85% vs. 11.07 ± 1.57%, *p* = 0.174; Fig 4).

**Fig 5:**
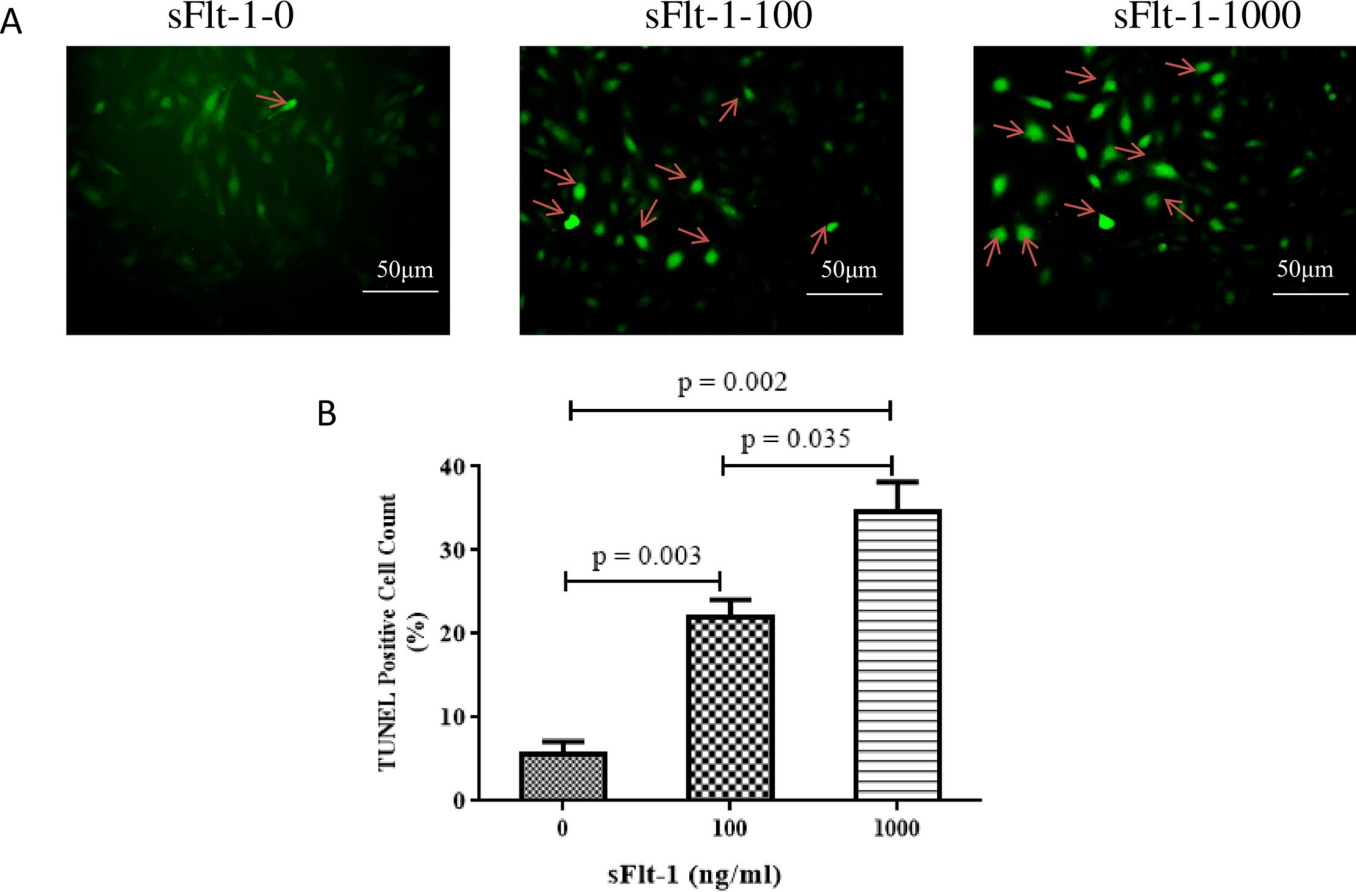
The apoptosis rate of HUVECs was measured using TUNEL assay. The figure showed a representative set of the graph of apoptosis rate of HUVECs by TUNEL staining. A: the indicated concentrations of sFlt-1-treated group, and B: statistical result of apoptosis rate of HUVECs in different groups. TUNEL analysis showed that sFlt-1 could induce an abundance of apoptosis in a dose-dependent manner (100 ng/ml vs. 0 ng/ml: 22.0 ± 3.60% vs. 5.67 ± 2.52%, *p* = 0.003; 1000 ng/ml vs. 0 ng/ml: 34.67 ± 6.03% vs. 5.67 ± 2.52%, *p* = 0.002; 1000 ng/ml vs. 100 ng/ml: 34.67 ± 6.03% vs. 22.0 ± 3.60%, *p* = 0.035). Results are the means ± standard deviation of three independent experiments. Scale bar denotes 50μm. Arrows indicate TUNEL-positive cells.

TUNEL analysis showed that sFlt-1 could induce an abundance of apoptosis in a dose-dependent manner (100 ng/ml vs. 0 ng/ml: 22.0 ± 3.60% vs. 5.67 ± 2.52%, *p* = 0.003; 1000 ng/ml vs. 0 ng/ml: 34.67 ± 6.03% vs. 5.67 ± 2.52%, *p* = 0.002; 1000 ng/ml vs. 100 ng/ml: 34.67 ± 6.03% vs. 22.0 ± 3.60%, *p* = 0.035; Fig 5).

### Mitochondrial apoptosis pathway in HUVECs was activated by sFlt-1

**Fig 6:**
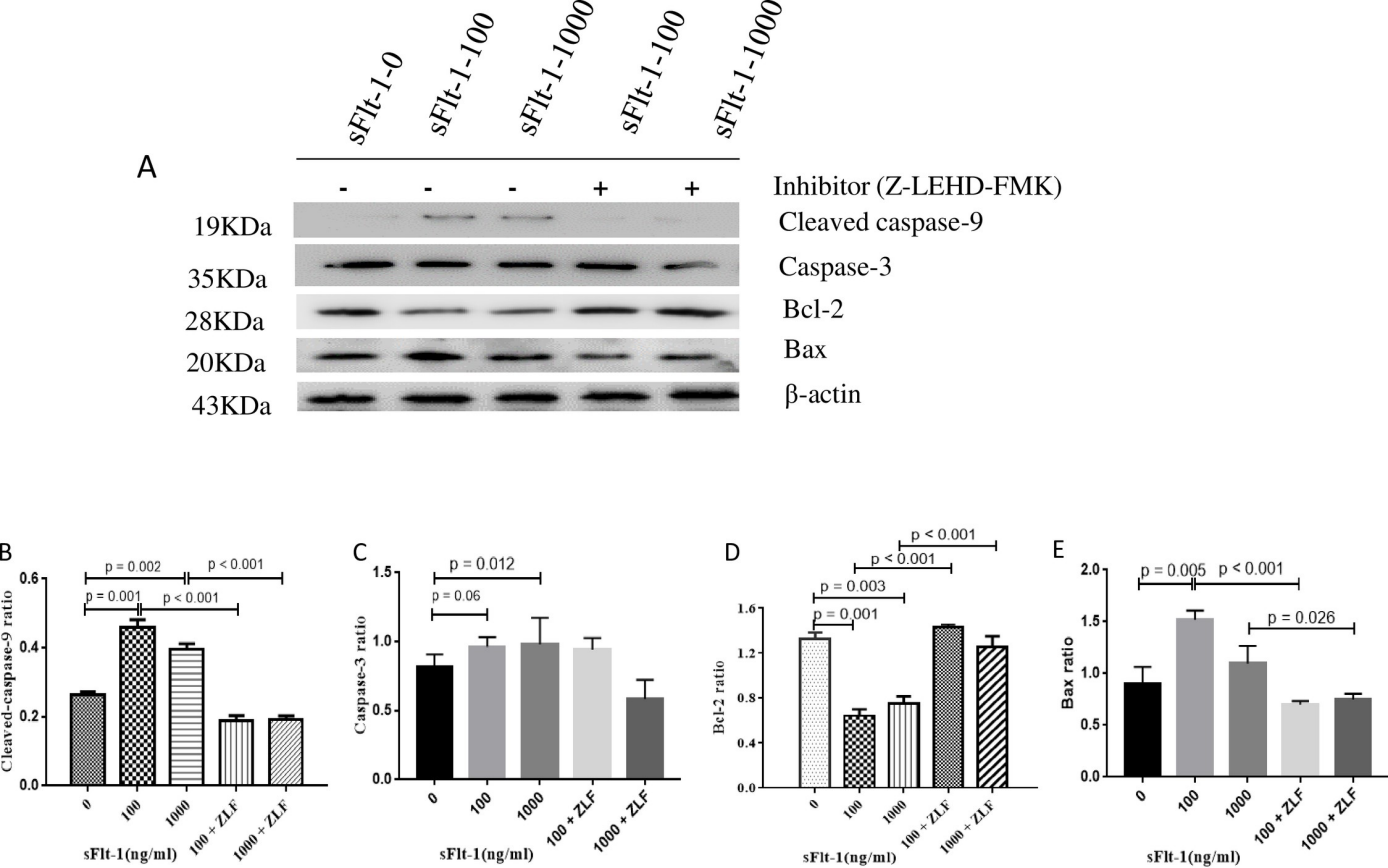
Expression of cleaved-caspase 9, caspase 3, Bcl-2 and Bax in sFlt-1-induced HUVECs with or without Z-LEHD-FMK incubation. The HUVECs were treated with the indicated concentrations of sFlt-1, and then treated with or without Z-LEHD-FMK. The protein expression levels of cleaved caspase-9, caspase-3, Bcl-2, Bax and β-actin were detected by western blotting (A). Relative protein levels of caspase-9 (B), caspase-3 (C), Bcl-2 (D) and Bax (E) versus β-actin are presented as histograms. Results are the means ± standard deviation of three independent experiments.

**Fig 7:**
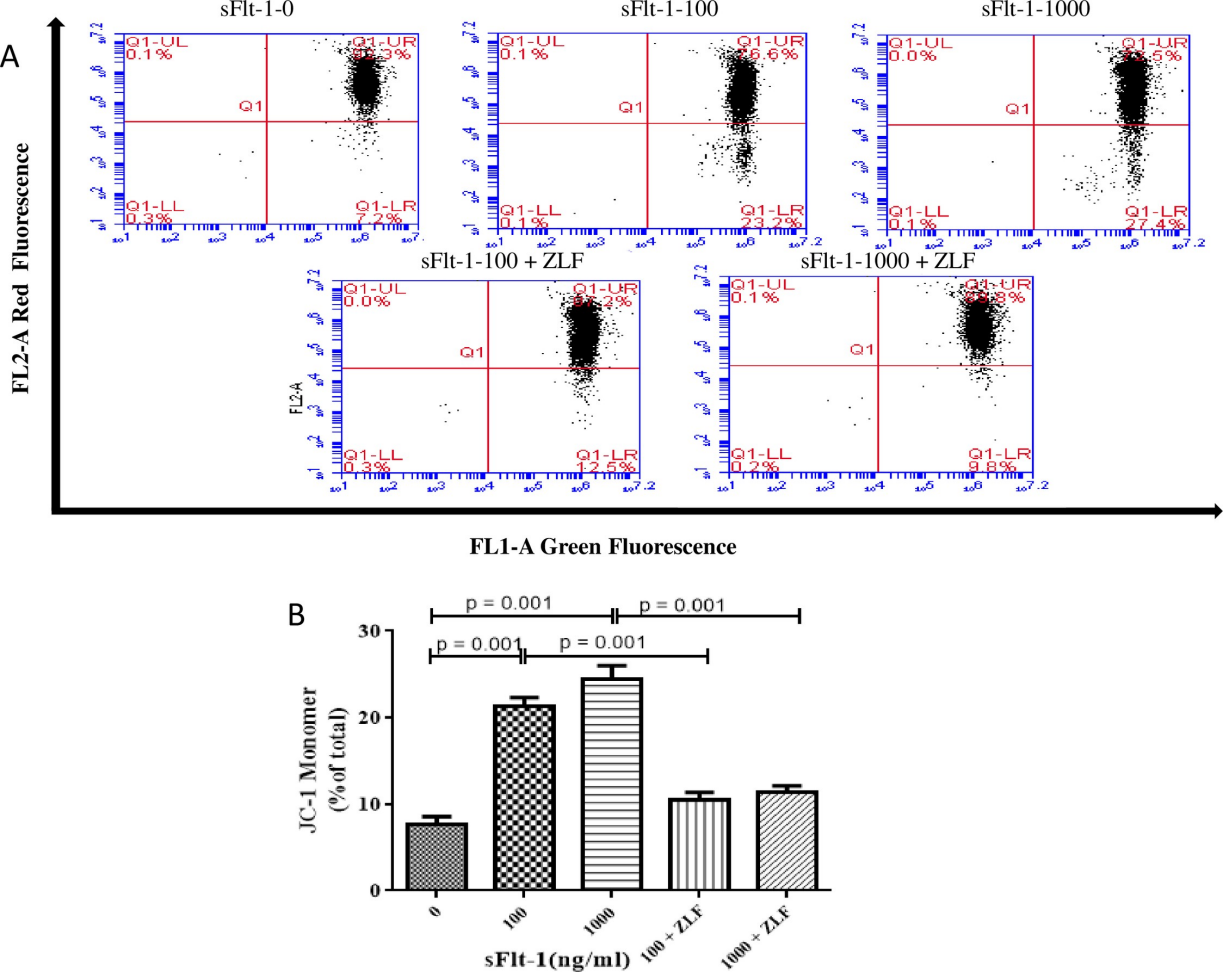
JC-1 monomer increased with the stimulation of sFlt-1 and decreased after administration of Z-LEHD-FMK in HUVECs. A: Effect of Z-LEHD-FMK on sFlt-1-induced disruption of mitochondrial membrane potential in HUVECs. B: statistical result of the percentage of JC-1 monomer in different groups. HUVECs with low ΔΨm increased from 7.63% in untreated cells to 21.33% in cells treated with 100 ng/ml sFlt-1, and further increased to 24.5% in cells with 1000 ng/ml sFlt-1. These effects were alleviated by addition of Z-LEHD-FMK.

**Fig 8:**
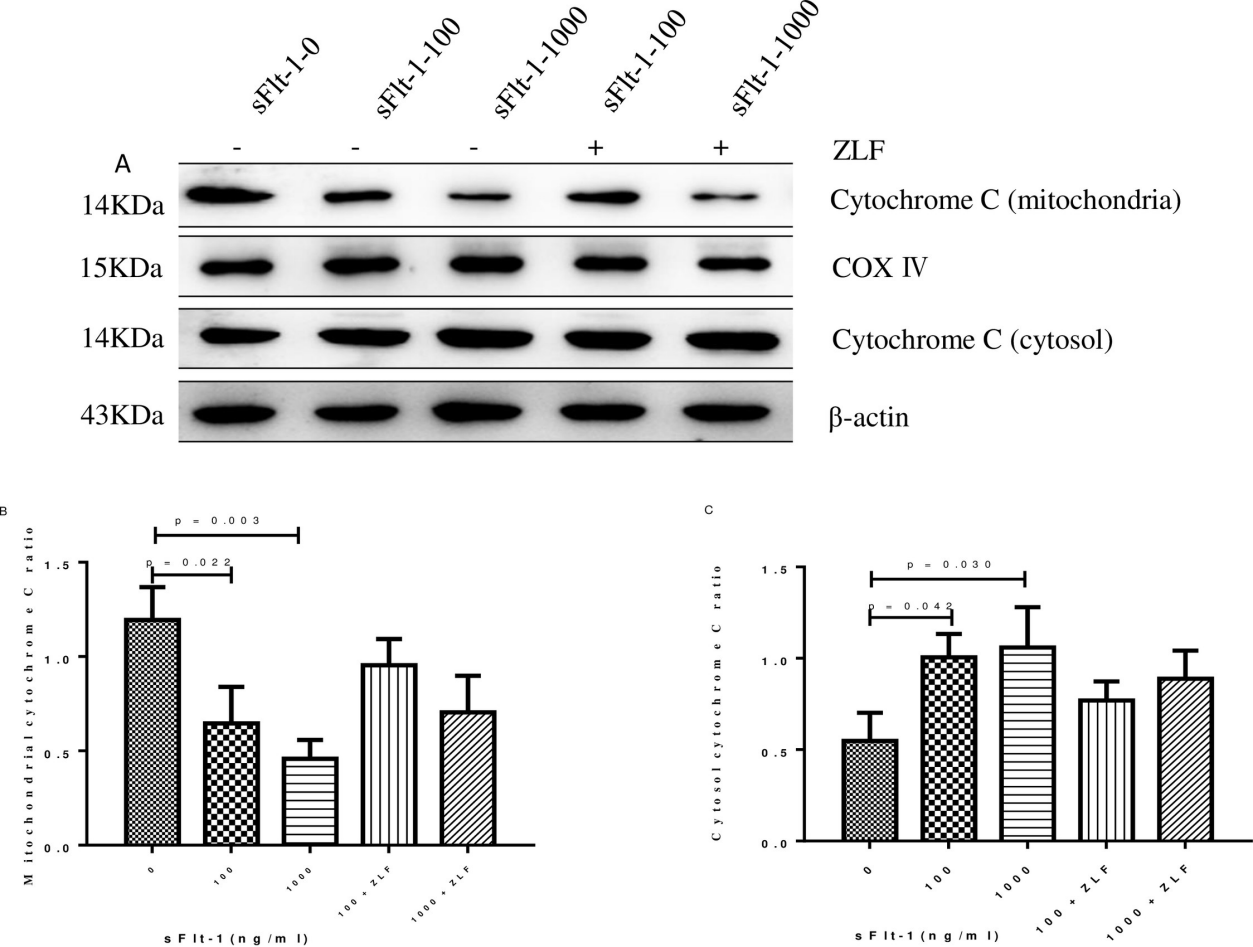
Cytochrome c expression in the mitochondria and cytosol in sFlt-1-induced HUVECs with or without Z-LEHD-FMK incubation. The HUVECs were treated with the indicated concentrations of sFlt-1, and then treated with or without Z-LEHD-FMK. The protein expression levels of cytochrome c expression in the mitochondria and cytosol were detected by western blotting (A). Relative protein levels in mitochondria (B) and cytosol (C) were presented as histograms. Results are the means ± standard deviation of three independent experiments.

In order to determine whether sFlt-1 induced activation of apoptosis pathway, we examined the changes in the activities of initiator caspase (cleaved caspase-9) and effector caspase (caspase-3) by Western blot. As shown in Fig 6, cleaved caspase-9 (100 ng/ml vs. 0 ng/ml: 0.46 ± 0.04 vs. 0.26 ± 0.01, *p* = 0.001; 1000 ng/ml vs. 0 ng/ml: 0.40 ± 0.03 vs. 0.26 ± 0.01, *p* = 0.002; Fig 6) and caspase-3 (100 ng/ml vs. 0 ng/ml: 1.11 ± 0.18 vs. 0.82 ± 0.09, *p* = 0.06; 1000 ng/ml vs. 0 ng/ml: 1.13 ± 0.08vs. 0.82 ± 0.09, *p* = 0.012; Fig 6) were clearly elevated after sFlt-1 stimulation. We next analyzed the changes in Bcl-2, Bax, Δψm, intracellular and mitochondrial cytochrome C in sFlt-1-treated HUVECs. As shown in Fig 6, the expression of Bcl-2 was down-regulated (100 ng/ml vs. 0 ng/ml: 0.64 ± 0.10 vs. 1.32 ± 0.10, *p* = 0.001; 1000 ng/ml vs. 0 ng/ml: 0.75 ± 0.11 vs. 1.32 ± 0.10, *p* = 0.003; Fig 6), whereas the expression of Bax was up-regulated (100 ng/ml vs. 0 ng/ml: 1.52 ± 0.09 vs. 0.90 ± 0.16, *p* = 0.005; 1000 ng/ml vs. 0 ng/ml: 1.10 ± 0.17 vs. 0.90 ± 0.16, *p* = 0.268; Fig 6). To investigate mitochondrial function, mitochondrial membrane potential was assayed. We found the HUVECs with low ΔΨm increased from 7.63% in untreated cells to 21.33% in cells treated with 100 ng/ml sFlt-1, and further increased to 24.5% in cells treated with 1000 ng/ml sFlt-1 (Fig 7). In addition, we examined the influence of sFlt-1 on the release of cytochrome C. The results showed a decrease in mitochondrial cytochrome C (100 ng/ml vs. 0 ng/ml: 0.64 ± 0.19 vs. 1.19 ± 0.17, p = 0.022; 1000 ng/ml vs. 0 ng/ml: 0.46 ± 0.10 vs. 1.19 ± 0.17, *p* = 0.003; Fig 8B) and an increase in the release of cytochrome C into the cytosol (100 ng/ml vs. 0 ng/ml: 1.00 ± 0.22 vs. 0.55 ± 0.15, *p* = 0.042; 1000 ng/ml vs. 0 ng/ml: 1.06 ± 0.22 vs. 0.55 ± 0.15, *p* = 0.030; Fig 8C). These results indicated that sFlt-1 might induce the activation of mitochondrial pathway leading to Bax/Bcl-2-mediated early apoptosis in HUVEC cells.

### Caspase-9 inhibitor Z-LEHD-FMK reduced sFlt-1-induced activation of mitochondrial apoptosis pathway

We determined the changes in mitochondrial-associated apoptotic molecules in HUVECs treated with Z-LEHD-FMK by Western blot. As the results showed, Z-LEHD-FMK could significantly decrease the expression of vWF (Fig 3). Obviously, a significant reduction in cleaved caspase-9 was observed when the Z-LEHD-FMK was added to sFlt-1 (Fig 6B). In addition, Z-LEHD-FMK could partially moderate the decrease of Bcl-2 (Fig 6D), alleviate the increase in the Bax (Fig 6E) and the ΔΨm (Fig 7), and inhibit the release of cytochrome C from mitochondria to cytoplasma induced by sFlt-1 (Fig 8). Taken together, these results indicated that endothelial dysfunction was associated with up-regulation of sFlt-1, which promoted HUVECs apoptosis via activation of mitochondrial pathway.

## Discussion

Plasma sFlt-1 levels were significantly elevated in IgAN patients and correlated with proteinuria, hypertension and vWF levels [12]. In our present study, we found pIgA1 complex derived from IgAN stimulation upregulated sFlt-1 levels in HUVECs, which further damaged the proliferation ability of HUVECs by triggering the mitochondrial apoptosis pathway. The proapoptotic effect was reversed by caspase 9 inhibitor. These results implicated sFlt-1 could lead to endothelial cell injury and finally destroy the glomerular filtration barrier, leading to the pathogenesis of IgAN.

Endothelial dysfunction, which is observed in a considerable proportion of patients with IgAN, is considered to be involved in the development and the progression of IgAN [5]. In the present study, we explored the correlation of elevated sFlt-1 with IgA complex, and founded that IgAN patients with high IgA complex levels had higher serum sFlt-1 levels. In addition, we detected the sFlt-1 levels in culture supernatant of HUVECs and observed elevated levels of sFlt-1 expression by pIgA1 complex derived from IgAN patients in a dose-dependent manner. However, no significant correlation was found between IgA-IgG complex and vWF levels. We next explored the correlations of elevated sFlt-1 with vascular injury biomarker. Patients with IgAN who had elevated sFlt-1 also had higher levels of vWF. And we found the expression of vWF was significantly upregulated by sFlt-1 stimulation. Our data also showed sFlt-1 protein could inhibit cell proliferation and induce apoptosis in HUVECs in a time and dose dependent manner, which was in accordance with previous reports [9, 17]. Combined with our previous results demonstrating a correlation between sFlt-1 and vWF levels in patients with IgAN, these results supported that sFlt-1 was a causal factor for endothelial injury in IgAN. The polymeric IgA1 complex appeared to act directly on sFlt-1, and the latter further stimulated the production of endothelial cells injury marker of vWF, contributing the endothelial dysfunction in IgAN.

The mechanism that the sFlt-1 leads to endothelial dysfunction is still not clear. Mitochondria is central integrator and transducer for apoptotic signals, which forms the nexus between the non-specific inducer phase and the final execution phase of apoptosis [18, 19]. A previous study by Wang et al. found that glomerular endothelial cells underwent apoptosis through the mitochondrial-dependent pathway [20]. Caspase-9 initiates the mitochondria-dependent apoptotic cascade by forming the apoptosome [21, 22]. To gain insight into the role of the sFlt-1-dependent apoptotic effect in HUVECs, we determined the increase in the levels of the caspase-9 active fragment and caspase-3. We observed sFlt-1 challenge could activate caspase-9 and caspase-3. These results indicated that sFlt-1 mediated apoptosis in HUVECs depending on cleaved caspase-9 active fragment and caspase-3.

The mitochondrial-dependent apoptosis pathway relies on the Bcl-2 family to regulate cell death [23, 24]. The Bcl-2 family controls the integrity of the outer mitochondrial membrane and is functionally divided into anti- and pro-apoptotic proteins. Bax forms proteolipid pores within the outer mitochondrial membrane, resulting in the loss of Δψm, the release of cytochrome c into the cytosol and activation of caspase-9 [25, 26]. In the present study, we observed that sFlt-1 increased the level of Bax, decreased Bcl-2 level, and facilitated cytochrome c release from mitochondria. In addition, our results revealed that mitochondrial membrane potential increased after sFlt-1 stimulation. These results demonstrated that sFlt-1 promoted HUVECs apoptosis through the mitochondrial apoptotic pathway.

The involvement of sFlt-1 in HUVECs was further confirmed by blocking experiments. Here, caspase 9 inhibitor, Z-LEHD-FMK, was administrated to verify our results. We found pretreatment of cells with the Z-LEHD-FMK significantly decreased the level of endothelial injury marker of vWF in the supernatant of HUVECs challenged by sFlt-1 protein. Z-LEHD-FMK inhibited sFlt-1-induced mitochondrial-mediated apoptosis, revealing that the increased Bcl-2, decreased Bax, cytochrome c release, caspase-9 activation, as well as ΔΨm. Taking the data in aggregate, the sFlt-1- induced -injury appears to act as an upstream signal which triggers mitochondrial pathway and promotes apoptosis in HUVECs. These findings suggest caspase 9 inhibitor can serve as a therapeutic strategy to attenuate sFlt-1 induced mitochondrial-dependent apoptosis of HUVECs.

In conclusion, our study demonstrated that sFlt-1 expression was up-regulated by the challenge of pIgA1 complex derived from patients with IgAN. Furthermore, increased sFlt-1 facilitated human umbilical vein endothelial cells apoptosis via activation of the mitochondrial-dependent pathway. This was the first study to highlight the important role of sFlt-1 in HUVECs apoptosis and provide a potential therapeutic target for the treatment of IgAN.

## Supporting information

S1 Raw images (PPTX)

## Abbreviations

IgAN: Immunoglobulin A nephropathy
sFlt-1: soluble fms-like tyrosine kinase-1
HUVECs: human umbilical vein endothelial cells
pIgA1: polymeric IgA1
vWF: von Willebrand Factor
VEGF: vascular endothelial growth factor
CKD: chronic kidney disease
CCK8: cell counting kit-8
TUNEL: Terminal deoxynucleotidyl transferase-dUTP nick-end labeling
