# Supplementary material for: The soluble VEGF receptor sFlt-1 contributes to endothelial dysfunction in IgA nephropathy
Source: PLoS One. 2020 Aug 13;15(8):e0234492. doi: 10.1371/journal.pone.0234492 (PMC7425938; doi:10.1371/journal.pone.0234492)
Supplement: S1 Raw images — (PPTX) [file pone.0234492.s001.pptx]

## Slide 1
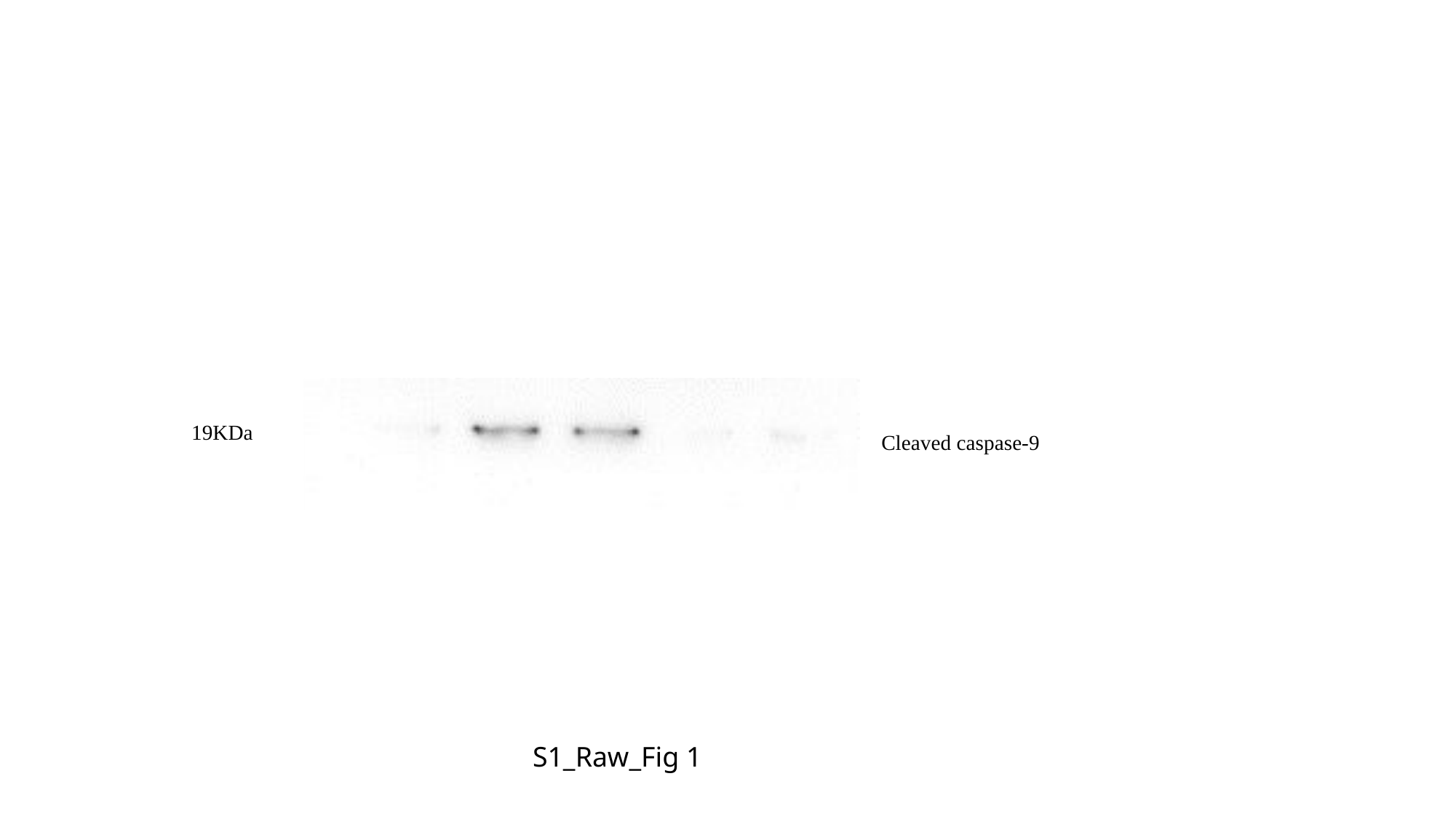

19KDa
 Cleaved caspase-9
S1_Raw_Fig 1

## Slide 2
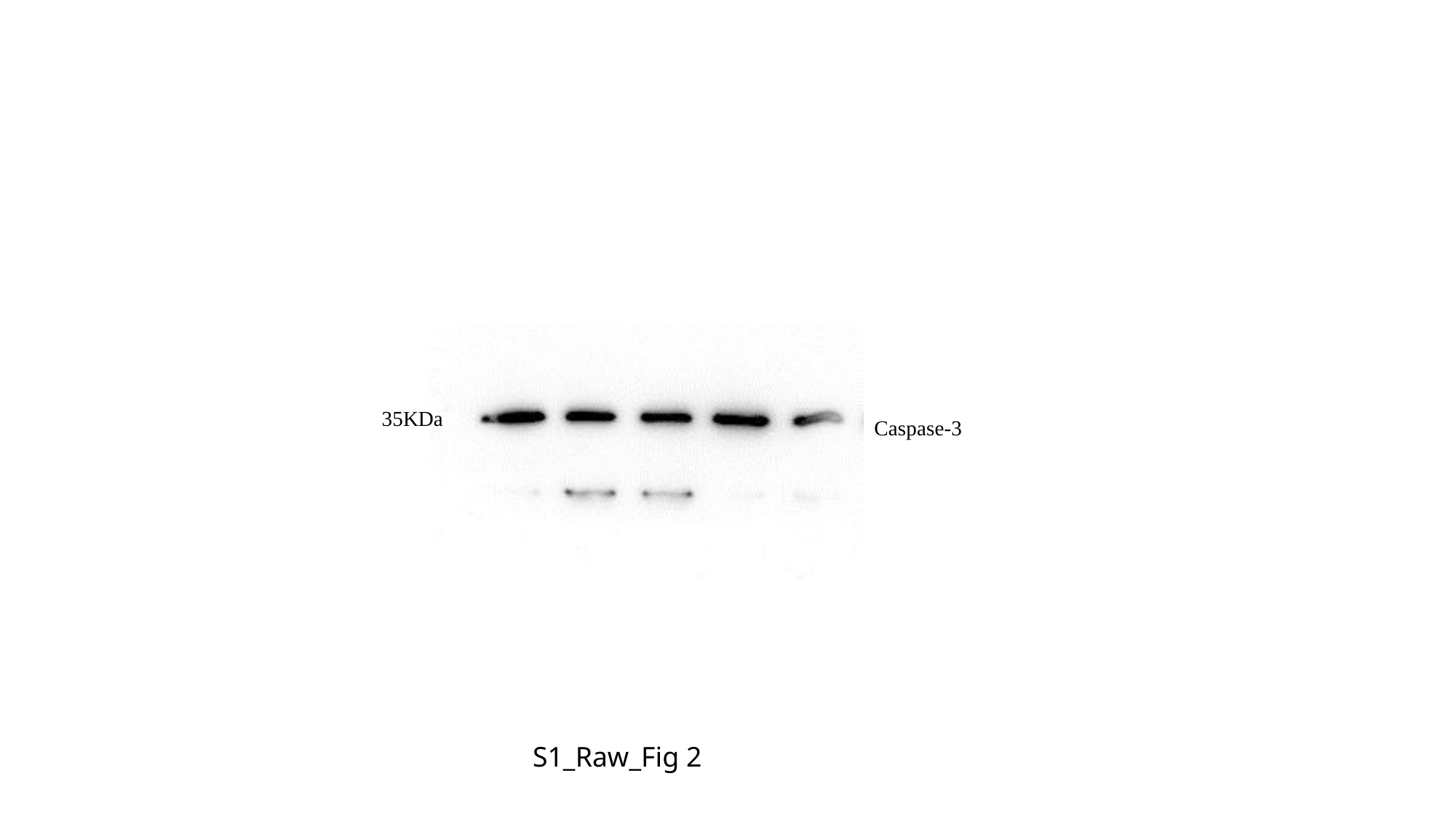

35KDa
Caspase-3
S1_Raw_Fig 2

## Slide 3
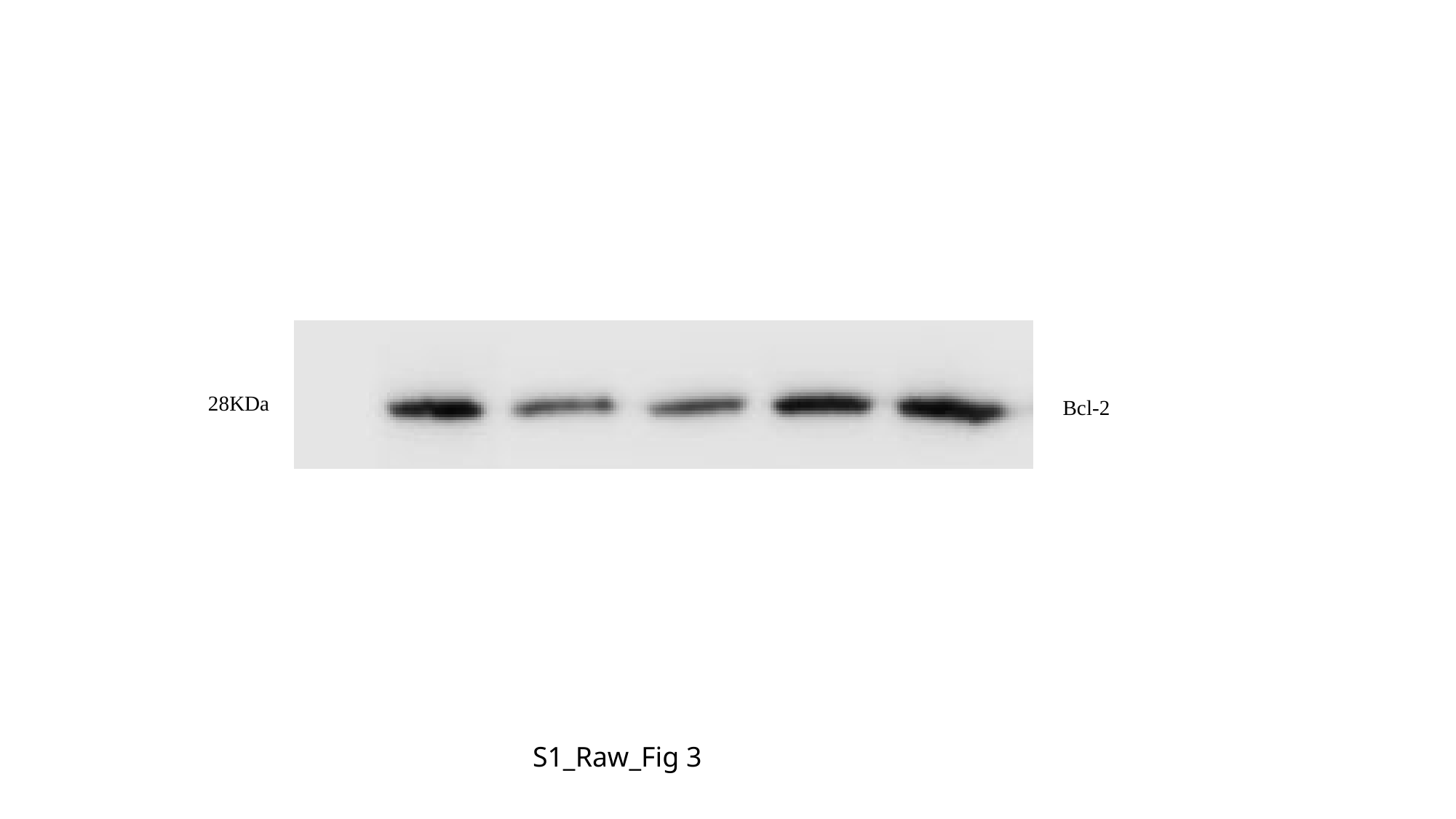

28KDa
Bcl-2
S1_Raw_Fig 3

## Slide 4
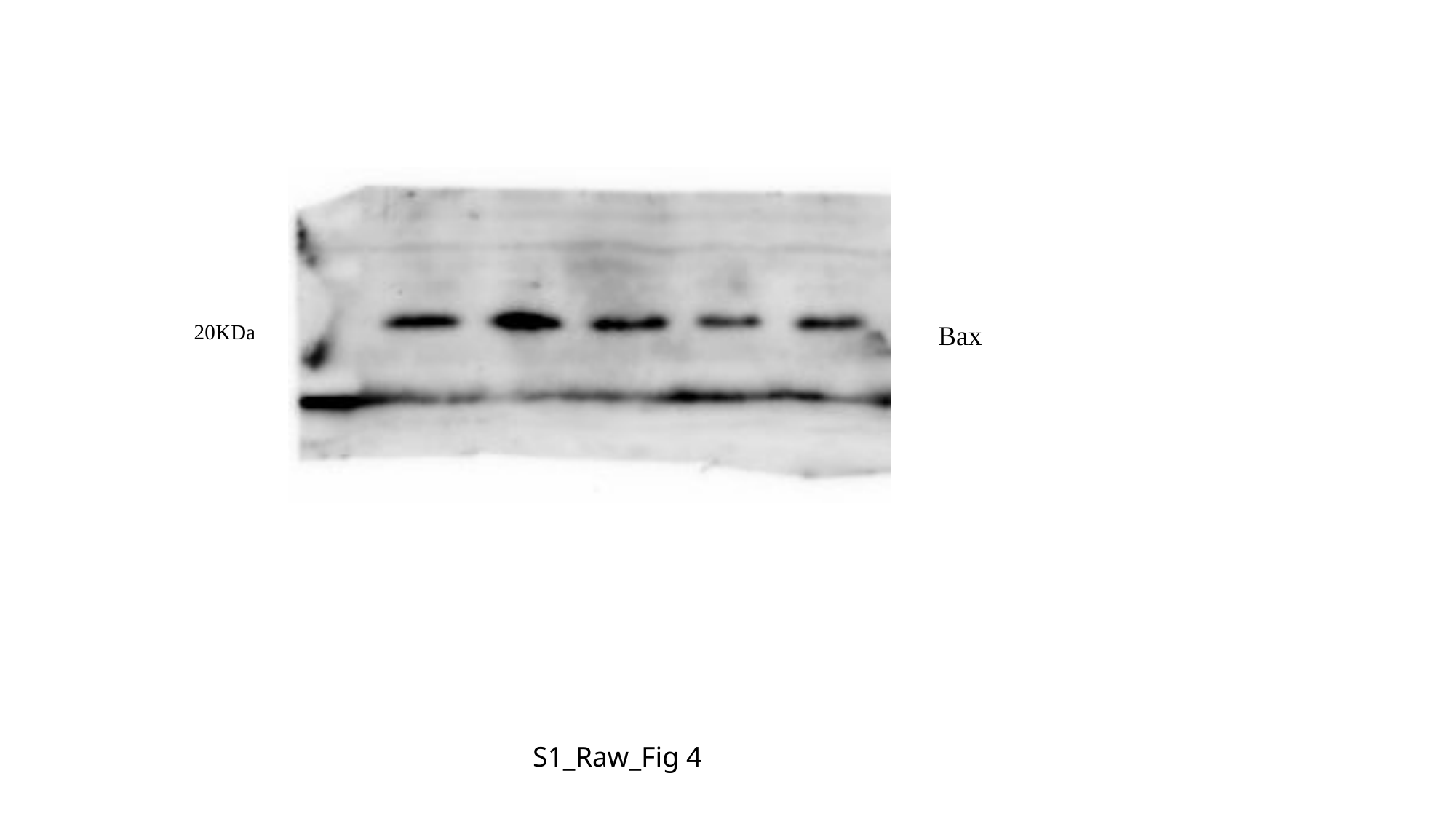

20KDa
Bax
S1_Raw_Fig 4

## Slide 5
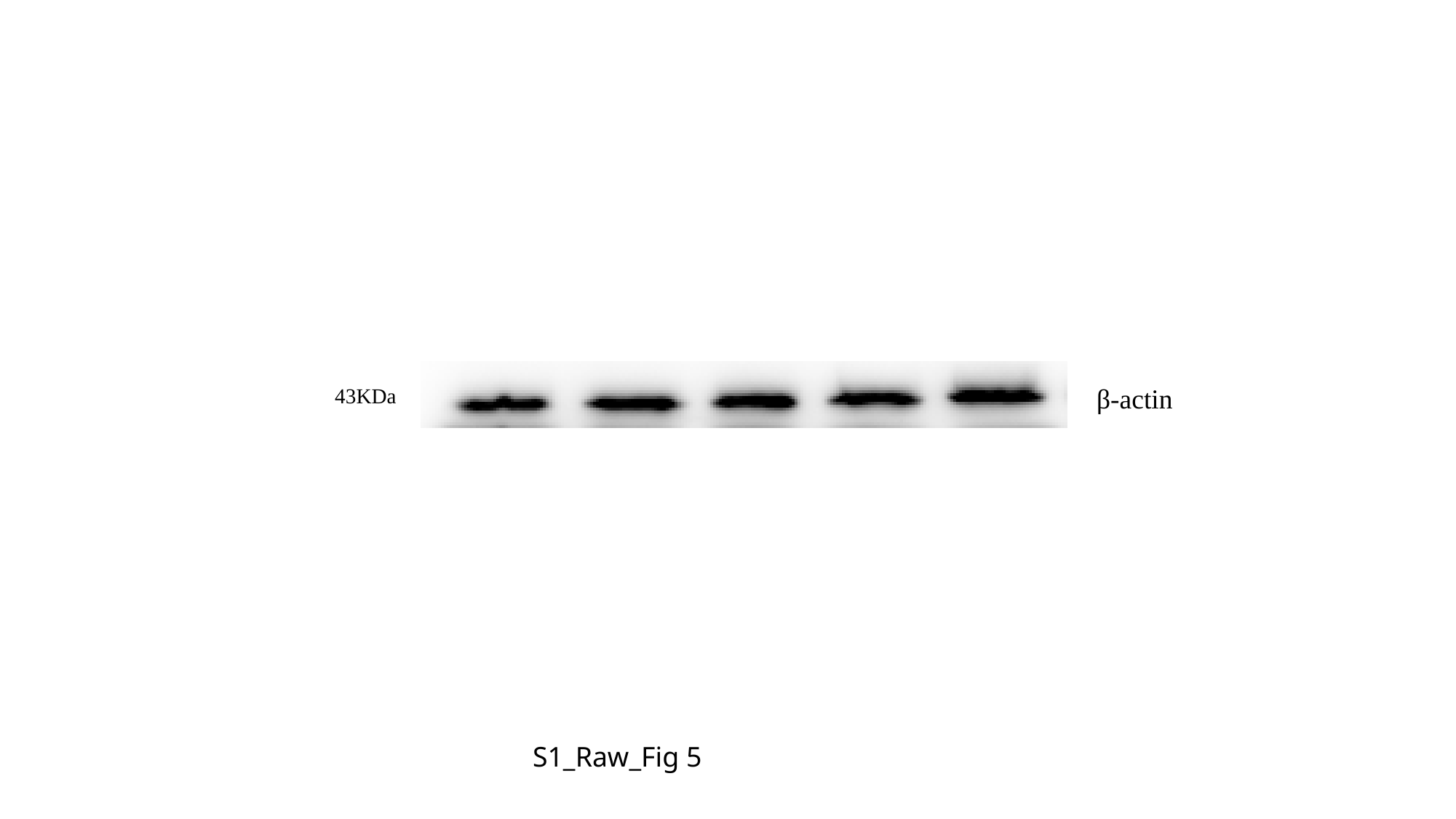

β-actin
43KDa
S1_Raw_Fig 5

## Slide 6
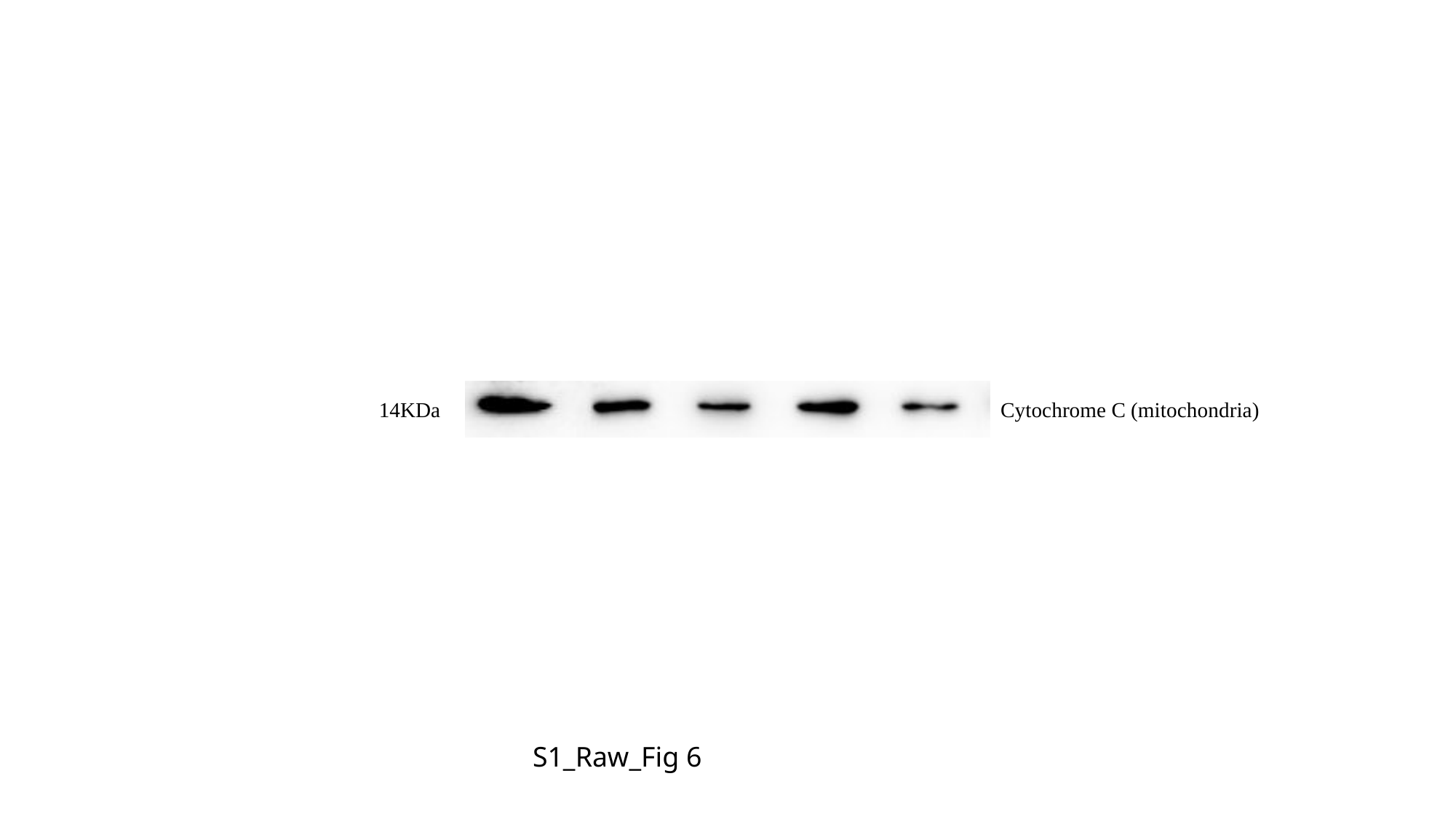

14KDa
Cytochrome C (mitochondria)
S1_Raw_Fig 6

## Slide 7
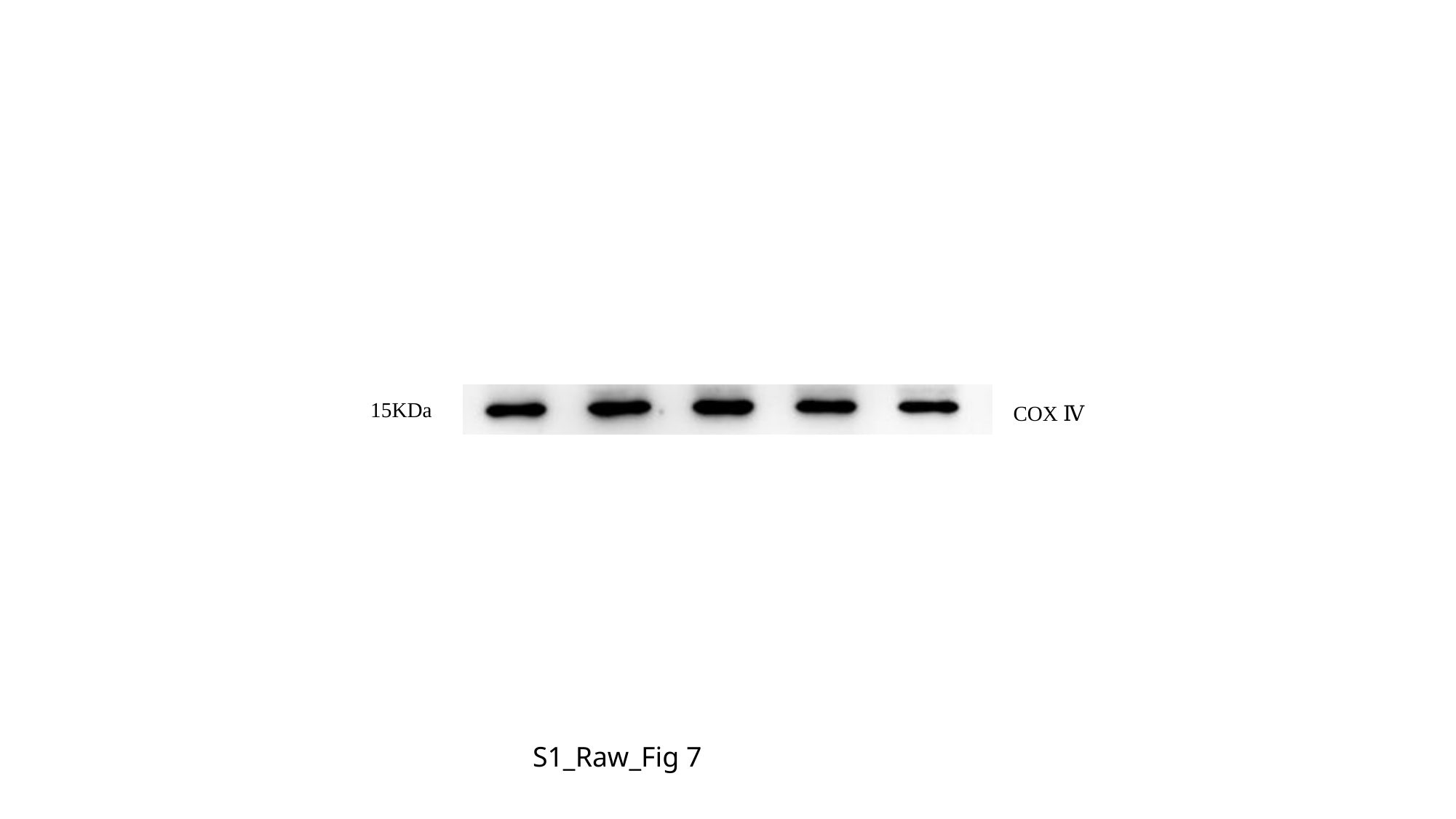

15KDa
COX Ⅳ
S1_Raw_Fig 7

## Slide 8
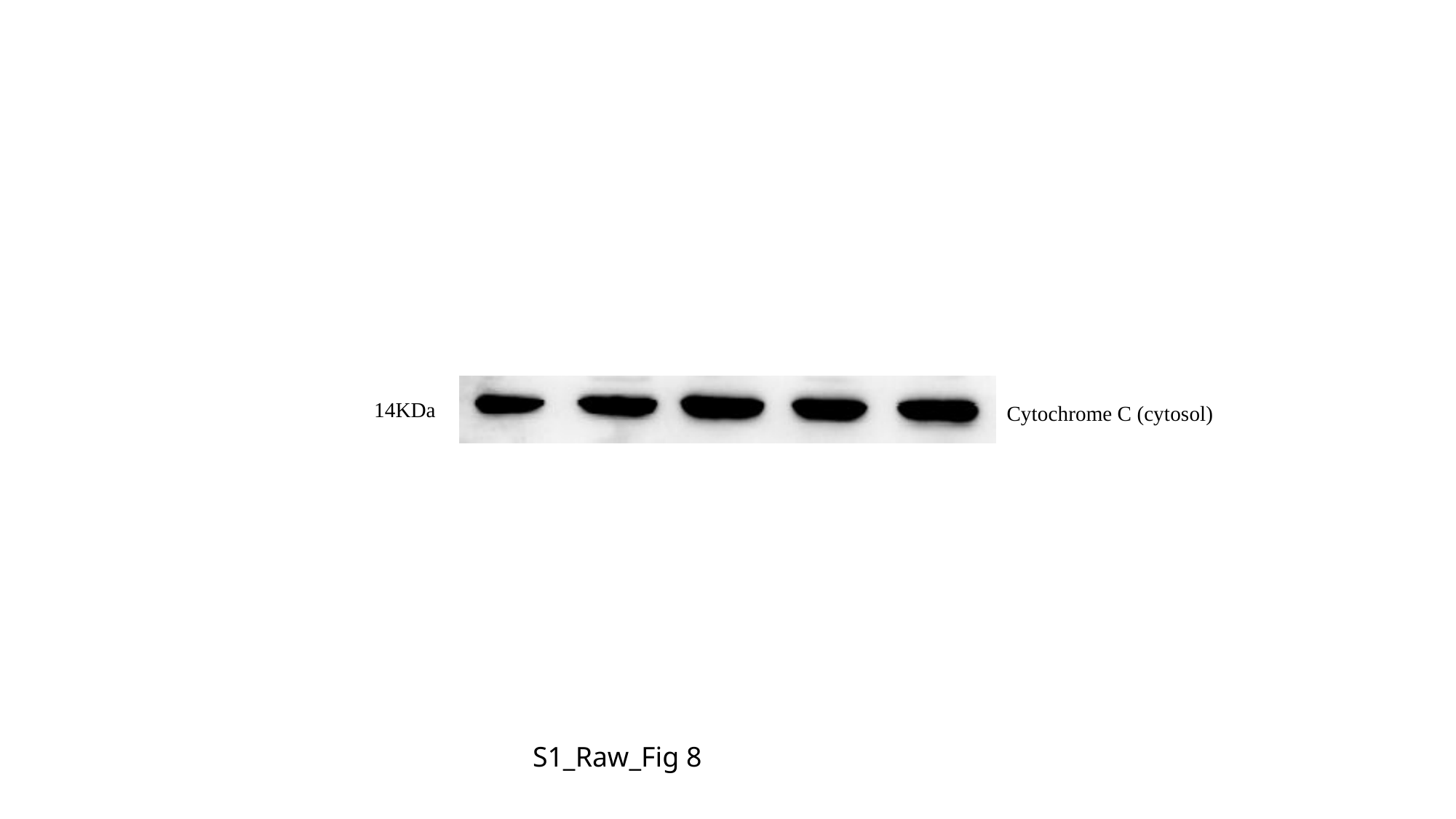

14KDa
Cytochrome C (cytosol)
S1_Raw_Fig 8
